# BMI-Associated Alleles Do Not Constitute Risk Alleles for Polycystic Ovary Syndrome Independently of BMI: A Case-Control Study

**DOI:** 10.1371/journal.pone.0087335

**Published:** 2014-01-31

**Authors:** Yvonne V. Louwers, Nigel W. Rayner, Blanca M. Herrera, Lisette Stolk, Christopher J. Groves, Thomas M. Barber, Andre G. Uitterlinden, Stephen Franks, Joop S. E. Laven, Mark I. McCarthy

**Affiliations:** 1 Department of Obstetrics and Gynecology, Subdivision of Reproductive Medicine, Erasmus MC University Medical Center, Rotterdam, the Netherlands; 2 Wellcome Trust Centre for Human Genetics, University of Oxford, Oxford, United Kingdom; 3 Department of Internal Medicine, Erasmus MC University Medical Center, Rotterdam, the Netherlands; 4 Oxford Centre for Diabetes, Endocrinology and Metabolism, University of Oxford, Oxford, United Kingdom; 5 Department of Epidemiology, Erasmus MC University Medical Center, Rotterdam, the Netherlands; 6 Institute of Reproductive and Developmental Biology, Imperial College London, Hammersmith Hospital, London, United Kingdom; University of Warwick – Medical School, United Kingdom

## Abstract

**Introduction:**

Polycystic Ovary Syndrome (PCOS) has a strong genetic background and the majority of patients with PCOS have elevated BMI levels. The aim of this study was to determine to which extent BMI-increasing alleles contribute to risk of PCOS when contemporaneous BMI is taken into consideration.

**Methods:**

Patients with PCOS and controls were recruited from the United Kingdom (563 cases and 791 controls) and The Netherlands (510 cases and 2720 controls). Cases and controls were of similar BMI. SNPs mapping to 12 BMI-associated loci which have been extensively replicated across different ethnicities, i.e., *BDNF*, *FAIM2*, *ETV5*, *FTO*, *GNPDA2*, *KCTD15*, *MC4R*, *MTCH2*, *NEGR1*, *SEC16B*, *SH2B1*, and *TMEM18,* were studied in association with PCOS within each cohort using the additive genetic model followed by a combined analysis. A genetic allelic count risk score model was used to determine the risk of PCOS for individuals carrying increasing numbers of BMI-increasing alleles.

**Results:**

None of the genetic variants, including *FTO* and *MC4R*, was associated with PCOS independently of BMI in the meta-analysis. Moreover, no differences were observed between cases and controls in the number of BMI-risk alleles present and no overall trend across the risk score groups was observed.

**Conclusion:**

In this combined analysis of over 4,000 BMI-matched individuals from the United Kingdom and the Netherlands, we observed no association of BMI risk alleles with PCOS independent of BMI.

## Introduction

Polycystic ovary syndrome (PCOS) is the most common endocrine disorder affecting up to 10% of females of reproductive age. [1] PCOS itself as well as its separate phenotypic characteristics demonstrate familial aggregation [2], [3] and its heritability has been estimated as high as 65%. [4] Key features of the syndrome include ovulatory dysfunction, hyperandrogenism and polycystic ovarian morphology. Moreover, the majority of patients with PCOS have overweight or obesity. [5] It has been well established that obesity worsens the phenotype of PCOS. Lifestyle interventions and weight-loss not only improve body composition and insulin resistance in patients with PCOS, they also ameliorate the reproductive phenotype.[6]–[8] Although this close relationship between PCOS and obesity clearly exists, underlying mechanisms are far from being understood.

Obesity is also known to be a highly heritable trait [9], [10] and its genetics have been widely and successfully investigated using genome wide association studies (GWASs).[11]–[15] Since patients with PCOS have increased BMI levels compared to controls, genetic variants influencing PCOS might well include BMI risk alleles such that GWAS signals identified influencing PCOS might in fact be driven by primary effects on BMI. Therefore, it is of importance to evaluate whether adjustment or matching for BMI would eradicate the potential for variants influencing BMI to have an apparent effect on PCOS risk. If these effects remain after taking case-control differences in BMI into consideration, it might suggest either that a single BMI measurement is not an adequate proxy for lifelong BMI when it comes to specifying the effects of BMI on PCOS, or that the BMI risk alleles have pleiotropic effects on BMI as well as PCOS. The latter has been suggested for SNPs mapping to the *FTO* gene in association with obesity and type 2 diabetes mellitus in Asians. [16], [17].

Previous studies observed association of risk-alleles mapping to the *FTO* and *MC4R* gene with PCOS and its phenotypic characteristics.[18]–[23] However, these studies did not include BMI-matched case-control sets and had relatively small sample sizes (number of cases ranging from 65 to 800 and less than 1000 controls).

Therefore, we studied twelve BMI-associated loci in BMI-matched case-control sets from two large university medical centers to determine the effect on PCOS-susceptibility independently of current BMI.

## Materials and Methods

### Ethics Statement

All clinical investigations were conducted according to the guidelines in the Declaration of Helsinki. The study was approved by the medical ethics committee from the Erasmus MC University Medical Centre. Approval for the UK study was obtained from the North Thames Multicenter Research Ethics Committee [MREC/99/2/45]). All subjects provided fully written informed consent.

### Subjects

Independent European PCOS populations from the United Kingdom (UK) and the Netherlands were included in this study. The UK case-control set included a total of 1354 women, of whom 563 were diagnosed with PCOS and 791 served as controls. The case-control set from the Netherlands consisted of 510 patients diagnosed with PCOS and 2720 control women from the general population. BMI levels between cases and controls in both studies were similar (p-value >0.05). Patients in both cohorts were diagnosed according to 2003 Rotterdam criteria. [24] In agreement with these criteria two of the following three symptoms should be present: oligo-ovulation and/or anovulation with gonadotropins levels within the normal limits, biochemical and/or clinical hyperandrogenism and polycystic morphology of the ovaries (PCOM). Oligomenorrhea was defined as a cycle length over 35 days and amenorrhea as absence of menstrual bleeding. Biochemical hyperandrogenism was determined by calculation of the Free Androgen Index (FAI) as: 100 x T (nmol/L)/SHBG (nmol/L). A FAI exceeding 4.5 was used as a cut-off. Clinical hirsutism was assessed using the modified Ferriman-Gallwey score and defined as an FG-score of at least 8. PCOM was assessed by transvaginal ultrasound and defined as the presence of at least 12 follicles in one or both ovaries and/or increased ovarian volume >10 ml. Exclusion criteria were presence of related disorders with similar presentations such as Cushing’s disease and congenital adrenal hyperplasia. The controls from the UK were population-based and recruited as part of the UK Blood Services (UKBS) set up by the Wellcome Trust Case Control Consortium (WTCCC). [25] Control women from the Netherlands were derived from the Rotterdam study, a population-based prospective cohort study. [26] In brief, this is a large population-based study of elderly subjects from a specific area near Rotterdam (Ommoord). All women aged 45 years or older at onset of menopause and with available DNA were included in the present analyses. These population-based control groups provided reference groups of allele frequencies which reflect the local general European population, rather than being control groups wherein PCOS specifically was excluded. Patients and controls were of European descent.

### Genotyping and Quality Control

Table S1 summarizes the studied SNPs mapping to BMI-associated loci as identified by Frayling et al [11], Loos et al. [13], Thorleifsson et al [14] and Willer et al. [15] These 12 loci were established as genome wide significant between the years 2007–2009 during the first waves of GWAS and have been replicated across several ethnic populations ever since. [27] The studied SNPs were the lead SNPs mapping to the BMI-associated loci, as described in the aforementioned papers.[11], [13]–[15] Genotyping was carried out using Taqman “on demand”-assays (Applied Biosystems, Warrington, UK). The genotyping success rate was >95%. The Rotterdam Study controls were genotyped using the Illumina 550k array and imputed using HapMap2 CEU reference panel. [28] Genotypes of six of the SNPs were derived from this data: rs4074134 mapping to *BDNF*, rs7138803 mapping to *FAIM2*, rs7647305 mapping to *ETV5*, rs10838738 mapping to *MC4R*, rs10913469 mapping to *SEC16B*, rs7498665 mapping to *SH2B1*. SNP rs11084753 had an imputation quality of 94%, rs6548238 of 95% and all other SNPs had an imputation quality of >99%. The other SNPs were genotyped using Taqman “on demand”-assays (Applied Biosystems, Warrington, UK). None of the SNPs deviated from Hardy-Weinberg equilibrium (HWE).

### Statistical Analysis and Power Calculation

Association analyses were initially carried out within each case-control set separately. The additive genetic model was tested using PLINK (v.1.07) [29] and IBM SPSS version 20 (IBM Statistical Package for the Sociological Sciences Inc., Chicago, USA). The combined effect of the BMI-increasing alleles in the two populations was evaluated using a fixed-effects meta-analysis in GWAMA [30] for SNPs with heterogeneity (I^2^) less than 25%. When I^2^ exceeded 25%, a random effect meta-analysis was performed using statistical software package R (http://www.r-project.org). Moreover, we studied the association of BMI-increasing alleles with PCOS when only including individuals with a BMI ≥30 kg/m^2^ and in a second analysis when only including individuals with a BMI <30 kg/m^2^.

Using Genetic Power Calculator software we determined that with the sample size of the total case control set (cases: n = 1073; controls: n = 3511), we reached approximately 95% power to detect association of a risk allele of frequency ≥0.2 having an odds ratio of ≥1.3 and an alpha of 0.05 (http://pngu.mgh.harvard.edu/~purcell/gpc/). [31] Since we selected the genetic variants, we did not correct for multiple testing and a *P* value <0.05 was considered statistically significant.

To test for the combined effect of all the BMI-associated alleles on PCOS susceptibility and to estimate the genetic risk of having PCOS for these women dependent on the number of BMI-increasing alleles present, we calculated the Genetic Risk Score (GRS). The GRS was modeled as a continuous variable and the calculation was carried out using R (http://www.r-project.org/). Using the GRS we assume that each SNP in the panel contributes equally to PCOS risk and that each individual allele has an equal and additive effect on risk. To obtain accurate counts of BMI-increasing alleles, only individuals with genotypes for at least 90% of SNPs (11 out of 12) were included. Based on this criterion, a total of 1264 individuals, i.e., 512 cases and 752 controls, from the UK and 3150 individuals from the Netherlands, i.e., 502 cases and 2648 controls, were included in the GRS-analysis. This method was described previously. [32], [33] Missing genotypes were replaced with the average risk score for each SNP in the total population. The maximum attainable score was 24 BMI-increasing alleles (12 SNPs * 2 alleles). The reference group was defined as 12 to 13 BMI-increasing alleles, which was the mean number of BMI-increasing alleles present in the controls. Analyses were carried out within the separate case-control sets as well as in the combined set. Finally, we calculated the overall trend across the GRS-groups using the Kruskal Wallis trend test (IBM SPSS version 20).

## Results

The UK cases had a mean BMI of 26.0 kg/m^2^ (±10.9 SD) and the controls had a BMI of 25.9 kg/m^2^ (±4.57 SD), whereas the cases from the Netherlands had a median BMI of 26.4 kg/m^2^ (IQR 22.4–31.7) and the controls had a BMI of 26.3 kg/m^2^ (IQR 23.97–29.1). The prevalence rates of normal weight, overweight en obese individuals are depicted in Table 1. Although the BMI levels of the total group of cases and controls were similar in the samples from the UK as well as the Netherlands, these prevalences were significantly different (*P*<0.001). Therefore, we also ran separate analyses including only individuals with BMI ≥30 kg/m^2^ and including only individuals with a BMI <30 kg/m^2^. Allele frequencies of the BMI-risk alleles in the cases and controls of both case-control sets are reported in Table S2.

**Table 1:** The prevalence rates of normal weight, overweight en obese patients with PCOS and controls.

		The United Kingdom		*P*	The Netherlands		*P*
		% cases	% controls		% cases	% controls	
Normal weight	BMI <25	42.7	50.3	<0.001	43.9	35.2	<0.001
Overweight	BMI ≥25 and <30	17.6	32.4		22.2	45.5	
Obese	BMI ≥30	39.7	17.3		33.9	19.3	

*BMI* Body Mass Index; *P* p-value.

First, we tested whether carrying BMI-associated alleles influenced PCOS susceptibility in cases and controls who were of similar BMI (Table 2). SNP rs7498665 mapping to the *SH2B1* locus was significantly associated with a decreased risk of having PCOS (OR  = 0.79; 95%CI: 0.69–0.90, *P*  = 0.001) in the case-control set from the Netherlands. No such association was evident in the case-control set from the UK (OR  = 1.04; 95% CI: 0.88–1.22, *P*  = 0.64) or in the random effects meta-analysis. Moreover, none of the other SNPs was significantly associated with PCOS, neither in the separate UK and Dutch analysis nor in the meta-analysis. The BMI-increasing alleles were not associated with PCOS in the meta-analysis including only individuals with BMI<30 kg/m^2^, nor were they associated with PCOS in the BMI ≥30 kg/m^2^ group (Table S3 and Table S4, respectively).

**Table 2:** Genetic association results for BMI-increasing risk alleles with PCOS in the United Kingdom and The Netherlands.

					United Kingdom			The Netherlands			Meta-analysis	
					cases n = 563 controls n = 791			cases n = 510 and control n = 2720			cases n = 1073 and control n = 3511	
SNP	CHR	position	Locus name	BMI-increasing risk allele	Overall frequencyrisk allele	OR per risk allele (95% CI)	*P*	Overall frequencyrisk allele	OR per risk allele (95% CI)	*P*	OR per risk allele (95% CI)	*P*
rs4074134	11	27603861	*BDNF*	G	0.79	1.10 (0.91–1.33)	0.34	0.79	0.86 (0.74–1.02)	0.08	0.97 (0.76–1.23)*	0.79
rs7138803	12	48533735	*FAIM2*	A	0.38	1.05 (0.90–1.23)	0.53	0.37	1.02 (0.89–1.17)	0.80	1.03 (0.93–1.14)	0.54
rs7647305	3	187316984	*ETV5*	C	0.78	1.09 (0.90–1.31)	0.38	0.80	0.96 (0.82–1.14)	0.66	1.01 (0.90–1.15)	0.82
rs9939609	16	52378028	*FTO*	A	0.42	1.17 (1.00–1.37)	0.05	0.36	1.01 (0.88–1.16)	0.87	1.08 (0.93–1.25)*	0.29
rs10938397	4	44877284	*GNPDA2*	G	0.45	0.99 (0.85–1.15)	0.87	0.42	1.15 (1.00–1.31)	0.05	1.06 (0.92–1.24)*	0.35
rs11084753	19	39013977	*KCTD15*	G	0.68	0.95 (0.80–1.11)	0.44	0.66	0.99 (0.86–1.14)	0.91	0.97 (0.87–1.08)	0.61
rs17782313	18	56002077	*MC4R*	C	0.24	1.06 (0.89–1.27)	0.51	0.25	1.12 (0.96–1.31)	0.14	1.09 (0.97–1.23)	0.13
rs10838738	11	47619625	*MTCH2*	G	0.36	1.00 (0.85–1.17)	0.99	0.33	1.09 (0.95–1.26)	0.20	1.05 (0.94–1.17)	0.37
rs2815752	1	72585028	*NEGR1*	A	0.60	0.99 (0.85–1.17)	0.93	0.61	1.04 (0. 91–1.19)	0.56	1.02 (0.92–1.13)	0.72
rs10913469	1	176180142	*SEC16B*	C	0.21	0.91 (0.75–1.11)	0.37	0.20	0.91 (0.77–1.08)	0.30	0.90 (0.80–1.03)	0.15
rs7498665	16	28790742	*SH2B1*	G	0.39	1.04 (0.88–1.22)	0.64	0.40	0.79 (0.69–0.90)	0.001	0.90 (0.73–1.18)*	0.45
rs6548238	2	624905	*TMEM18*	C	0.84	1.05 (0.85–1.30)	0.64	0.83	1.00 (0.83–1.19)	0.95	1.02 (0.89–1.17)	0.77

* random effect meta-analysis (I^2^>25%), otherwise fixed effect meta-analysis was performed. *CHR* chromosome, *SNP* single nucleotide polymorphism, *OR* odds ratio, *CI* confidence interval, *P* p-value.

**Figure 1:**
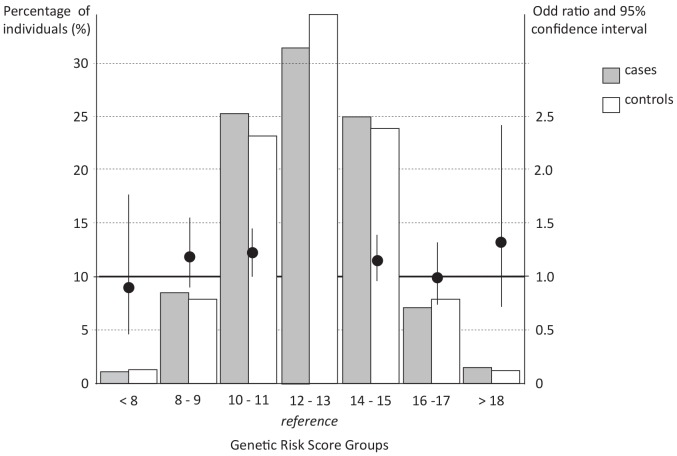
Combined impact of risk alleles on the risk of having PCOS compared to the reference risk group. Along the X-axis the risk categories are shown based on the number of BMI-increasing alleles. The histogram (Y-axis on the left) indicates the percentage of individuals for each risk-score group. The odds ratio and confidence intervals calculated based on the risk of having PCOS compared to the reference risk group are plotted on the Y axis on the right.

To determine whether the overall burden of BMI-increasing alleles was associated with PCOS status in these samples, a GRS was constructed (Figure 1, Table 3). The individuals carrying less than 8 BMI-associated alleles and individuals carrying over 18 BMI-associated alleles together account for a very small proportion of the total population, i.e., 2.6%. None of the GRS-groups was attributed more often to patients with PCOS compared with the reference GRS-group. Moreover, no overall trend in carrying an increasing number of BMI-associated alleles on PCOS susceptibility was observed neither for the total case-control set (*P*  = 0.44), nor for the separate case-control sets from the UK (P  = 0.97) or the Netherlands (*P*  = 0.17).

**Table 3:** Combined impact of BMI-increasing alleles on the risk of having PCOS.

	United Kingdom			The Netherlands			Combined		
GRS	Casesn (%)	Controlsn (%)	OR (95%CI)	Casesn (%)	Controlsn (%)	OR (95%CI)	Cases n (%)	Controlsn (%)	OR (95%CI)
**<8**	7 (1.4)	14 (1.9)	0.73 (0.29–1.86)	4 (0.8)	31 (1.2)	0.82 (0.28–2.34)	11 (1.1)	45 (1.3)	0.90 (0.46–1.76)
**8 to 9**	49 (9.6)	78 (10.4)	0.92 (0.61–1.38)	37 (7.4)	191 (7.2)	1.22 (0.83–1.81)	86 (8.5)	269 (7.9)	1.18 (0.90–1.55)
**10 to 11**	121 (23.6)	177 (23.5)	1.00 (0.74–1.36)	136 (27.1)	611 (23.1)	1.41 (1.09–1.81)	257 (25.3)	788 (23.2)	1.20 (1.00–1.45)
**12 to 13**	173 (33.8)	254 (33.8)	*Reference*	146 (29.1)	922 (34.8)	*Reference*	319 (31.5)	1176 (34.6)	*Reference*
**14 to 15**	125 (24.4)	171 (22.7)	1.07 (0.79–1.45)	129 (25.7)	641 (24.2)	1.27 (0.98–1.64)	254 (25.0)	812 (23.9)	1.15 (0.96–1.39)
**16 to 17**	32 (6.3)	52 (6.9)	0.90 (0.56–1.46)	40 (8.0)	216 (8.2)	1.17 (0.80–1.71)	72 (7.1)	268 (7.9)	0.99 (0.74–1.32)
**>18**	5 (1.0)	6 (0.8)	1.22 (0.37–4.01)	10 (2.0)	36 (1.4)	1.75 (0.85–3.61)	15 (1.5)	42 (1.2)	1.32 (0.72–2.41)

Data are presented for pools of BMI-increasing alleles as odds ratio and confidence intervals. Only individuals with data of >90% of the SNPs available were included. The mean number of BMI-increasing alleles in the controls was used as the reference group. *GRS* genetic risk score; *n* number of individuals; *OR* odds ratio.

## Discussion

In this combined analysis including >4,000 patients with PCOS and controls from the United Kingdom and the Netherlands, we observed no association of genetic BMI-risk loci with PCOS when contemporaneous BMI is similar in cases and controls.

The last two years, genome wide association studies (GWASs) have emerged to identify genetic risk factors for PCOS. Two large studies identifying such PCOS-susceptibility loci in Han-Chinese patients have been published [34], [35] while GWAS in patients from Northern European ancestry are in progress. In the current study, we observed no systematic effect of the BMI-associated alleles on PCOS susceptibility in our BMI-matched case-control set. This infers that adjustment or matching for BMI will disentangle BMI-associated genetic signals to show up in PCOS GWAS and seems therefore not a genuine concern in the previous and upcoming GWASs in PCOS.

Presence of an increasing number of BMI-raising alleles is associated with an increased genetic predisposition to obesity. To determine the overall burden of BMI-associated alleles on risk for PCOS we calculated a counted genetic risk score and compared PCOS risk across such BMI risk groups. By doing so, we assumed that each allele has an equal and additive effect on PCOS risk. In practice some SNPs will have stronger effects than others. However, when the ORs are small as they are in our study, using a counted genetic risk model is appropriate. [32], [36] None of the GRS-groups was attributed more often to the patients diagnosed with PCOS than controls compared to the reference GRS-group. Moreover, no overall trend across the consecutive GRS groups was observed, strengthening the results from the allelic-association analysis that BMI-associated alleles seem not to have pleotropic effects on PCOS risk. Increased BMI levels and weight in PCOS seem to be mediated by other genetic factors determining an individual’s susceptibility to become obese or through modifying environmental effects. It has been shown that although women with PCOS reported a better, more healthy, dietary intake they seem to have an increased energy intake in combination with an increased sitting time without any discernible differences in total physical activity compared to women without PCOS. [37] Moreover, also in patients with PCOS, higher energy intake and glycemic index and lower physical activity, as well as age, smoking, alcohol intake, occupation, education and country of birth, were independently associated with BMI. [38].

In the current study we included population-based controls. Selection of controls in genetic studies is an extensively discussed issue. The main argument for using so-called ‘super controls’, i.e., individuals who are known to be unaffected, instead of population-based controls, is that the power is expected to improve because the difference in allele frequency between cases and controls is increased. For a disease as frequent as PCOS this would indeed increase the power to detect effects. However, only small samples of control women for whom PCOS is excluded are available. Moreover, these women are selected based on other criteria, for example tubal infertility. Consequently, it is more feasible to use a larger sample which is randomly selected from the general population as controls [39]–[41] and thereby compensating for the fact that we might also have also included women diagnosed with PCOS.

A potential limitation of the current study is that we matched BMI based on a single measurement. In general the BMI of men as well as in women increases throughout life. Therefore, a single BMI measurement may not be an appropriate proxy for lifetime BMI and might be a poor estimate of the long-standing effects of BMI on PCOS risk. However, since we observed no systematic effect of BMI-associated alleles on PCOS risk in our matched case-control set, this seems not to have influenced our results tremendously. It has been previously observed that the association of genetic variants in *FTO* and *MC4R* with BMI and weight strengthen during childhood up to age 20 years and then become weaker with increasing age during adulthood. [42] Therefore, as has been suggested for phenotypes associated with type 2 diabetes mellitus [17], longitudinal studies are needed to adequately explore the complex and dynamic nature of BMI-associated alleles on cardiometabolic characteristics in PCOS.

In conclusion, we have shown in two independent large PCOS case-control sets matched for BMI, that there is no systematic effect of BMI-associated alleles on PCOS risk suggesting that these alleles do not have a pleiotropic effect on PCOS susceptibility. Hence, adjusting for BMI in PCOS case-control GWAS studies should be an effective strategy for removing confounding effects of BMI on the association of other genetic variants and PCOS.

## Supporting Information

Table S1 **Studied BMI-associated loci.** *SNP* Single Nucleotide Polymorphism.
(DOC)

Table S2 **Allele frequencies in cases and controls from the United Kingdom and the Netherlands.** *SNP* Single Nucleotide Polymorphism.
(DOC)

Table S3 **Genetic association results for BMI-increasing risk alleles with PCOS in the United Kingdom and The Netherlands including non-obese cases and controls (BMI <30 kg/m^2^).** * random effect meta-analysis (I^2^>25%), otherwise fixed effect meta-analysis was performed. *chr* chromosome, *SNP* single nucleotide polymorphism, *OR* odds ratio, *CI* confidence interval, *P* p-value.
(DOC)

Table S4 **Genetic association results for BMI-increasing risk alleles with PCOS in the United Kingdom and The Netherlands including obese cases and controls (BMI ≥30 kg/m^2^).** * random effect meta-analysis (I^2^>25%), otherwise fixed effect meta-analysis was performed. *chr* chromosome, *SNP* single nucleotide polymorphism, *OR* odds ratio, *CI* confidence interval, *P* p-value.
(DOC)
