# Supplementary material for: BMI-Associated Alleles Do Not Constitute Risk Alleles for Polycystic Ovary Syndrome Independently of BMI: A Case-Control Study
Source: PLoS One. 2014 Jan 31;9(1):e87335. doi: 10.1371/journal.pone.0087335 (PMC3909077; doi:10.1371/journal.pone.0087335)
Supplement: Table S1 — Studied BMI-associated loci. SNP Single Nucleotide Polymorphism. (DOC) [file pone.0087335.s001.doc]

| **nearest gene** | **SNP** | **chromosome** | **position** | **paper in which the SNP was first cited** |
| --- | --- | --- | --- | --- |
| *BDNF* | rs4074134 | 11 | 27603861 | Thorleifsen et al |
| *FAIM2* | rs7138803 | 12 | 48533735 | Thorleifsen et al |
| *ETV5* | rs7647305 | 3 | 187316984 | Thorleifsen et al |
| *FTO* | rs9939609 | 16 | 52378028 | Frayling et al |
| *GNPDA2* | rs10938397 | 4 | 44877284 | Willer et al |
| *KCTD15* | rs11084753 | 19 | 39013977 | Willer et al |
| *MC4R* | rs17782313 | 18 | 56002077 | Willer et al |
| *MTCH2* | rs10838738 | 11 | 47619625 | Loos et al |
| *NEGR1* | rs2815752 | 1 | 72585028 | Willer et al |
| *SEC16B* | rs10913469 | 1 | 176180142 | Thorleifsen et al |
| *SH2B1* | rs7498665 | 16 | 28790742 | Willer et al |
| *TMEM18* | rs6548238 | 2 | 624905 | Willer et al |
