# Supplementary material for: BMI-Associated Alleles Do Not Constitute Risk Alleles for Polycystic Ovary Syndrome Independently of BMI: A Case-Control Study
Source: PLoS One. 2014 Jan 31;9(1):e87335. doi: 10.1371/journal.pone.0087335 (PMC3909077; doi:10.1371/journal.pone.0087335)
Supplement: Table S2 — Allele frequencies in cases and controls from the United Kingdom and the Netherlands. SNP Single Nucleotide Polymorphism. (DOC) [file pone.0087335.s002.doc]

|  | | | | **United Kingdom** | | | | **the Netherlands** | | | |
| --- | --- | --- | --- | --- | --- | --- | --- | --- | --- | --- | --- |
|  | | | | **cases** | | **controls** | | **cases** | | **controls** | |
| **SNP** | **nearest gene** | **allele 1** | **allele 2** | **frequency allele 1** | **frequency allele 2** | **frequency allele 1** | **frequency allele 2** | **frequency allele 1** | **frequency allele 2** | **frequency allele 1** | **frequency allele 2** |
| rs4074134 | *BDNF* | G | A | 0.80 | 0.20 | 0.78 | 0.22 | 0.77 | 0.23 | 0.80 | 0.20 |
| rs7138803 | *FAIM2* | G | A | 0.61 | 0.39 | 0.62 | 0.38 | 0.62 | 0.38 | 0.62 | 0.38 |
| rs7647305 | *ETV5* | C | T | 0.79 | 0.21 | 0.77 | 0.23 | 0.79 | 0.21 | 0.80 | 0.20 |
| rs9939609 | *FTO* | T | A | 0.54 | 0.46 | 0.58 | 0.42 | 0.62 | 0.38 | 0.63 | 0.37 |
| rs10938397 | *GNPDA2* | A | G | 0.54 | 0.46 | 0.54 | 0.46 | 0.55 | 0.45 | 0.59 | 0.41 |
| rs11084753 | *KCTD15* | G | A | 0.66 | 0.34 | 0.67 | 0.33 | 0.66 | 0.34 | 0.66 | 0.34 |
| rs17782313 | *MC4R* | T | C | 0.75 | 0.25 | 0.76 | 0.24 | 0.73 | 0.27 | 0.75 | 0.25 |
| rs10838738 | *MTCH2* | A | G | 0.64 | 0.36 | 0.64 | 0.36 | 0.64 | 0.36 | 0.67 | 0.33 |
| rs2815752 | *NEGR1* | A | G | 0.60 | 0.40 | 0.61 | 0.39 | 0.61 | 0.39 | 0.60 | 0.40 |
| rs10913469 | *SEC16B* | T | C | 0.80 | 0.20 | 0.79 | 0.21 | 0.81 | 0.19 | 0.79 | 0.21 |
| rs7498665 | *SH2B1* | A | G | 0.61 | 0.39 | 0.62 | 0.38 | 0.63 | 0.37 | 0.58 | 0.42 |
| rs6548238 | *TMEM18* | C | T | 0.85 | 0.15 | 0.84 | 0.16 | 0.83 | 0.17 | 0.83 | 0.17 |
