# Supplementary material for: BMI-Associated Alleles Do Not Constitute Risk Alleles for Polycystic Ovary Syndrome Independently of BMI: A Case-Control Study
Source: PLoS One. 2014 Jan 31;9(1):e87335. doi: 10.1371/journal.pone.0087335 (PMC3909077; doi:10.1371/journal.pone.0087335)
Supplement: Table S4 — Genetic association results for BMI-increasing risk alleles with PCOS in the United Kingdom and The Netherlands including obese cases and controls (BMI ≥30 kg/m2). * random effect meta-analysis (I2>25%), otherwise fixed effect meta-analysis was performed. chr chromosome, SNP single nucleotide polymorphism, OR odds ratio, CI confidence interval, P p-value. (DOC) [file pone.0087335.s004.doc]

|  | | | | | **United Kingdom** | | | **The Netherlands** | | | **Meta-analysis** | |
| --- | --- | --- | --- | --- | --- | --- | --- | --- | --- | --- | --- | --- |
| **SNP** | **chr** | **position** | **Locus name** | **BMI-increasing risk allele** | **Overall frequency risk allele** | **OR per risk allele (95% CI)** | ***P*** | **Overall frequency risk allele** | **OR per risk allele (95% CI)** | ***P*** | **OR per risk allele (95% CI)** | ***P*** |
| rs4074134 | 11 | 27603861 | *BDNF* | G | 0.80 | 1.04 (0.70-1.54) | 0.84 | 0.80 | 0.76 (0.57-1.01) | 0.56 | 0.86 (0.63-1.17) | 0.34* |
| rs7138803 | 12 | 48533735 | *FAIM2* | A | 0.38 | 1.06 (0.76-1.47) | 0.73 | 0.38 | 0.89 (0.67-1.12) | 0.28 | 0.95 (0.78-1.16) | 0.63 |
| rs7647305 | 3 | 187316984 | *ETV5* | C | 0.79 | 1.12 (0.76-1.65) | 0.55 | 0.81 | 0.88 (0.65-1.20) | 0.44 | 0.97 (0.76-1.23) | 0.78 |
| rs9939609 | 16 | 52378028 | *FTO* | A | 0.47 | 1.03 (0.74-1.41) | 0.87 | 0.38 | 0.87 (0.68-1.12) | 0.28 | 0.93 (0.76-1.13) | 0.45 |
| rs10938397 | 4 | 44877284 | *GNPDA2* | G | 0.48 | 1.03 (0.75-1.14) | 0.85 | 0.43 | 1.02 (0.80-1.30) | 0.87 | 1.03 (0.88-1.20) | 0.75 |
| rs11084753 | 19 | 39013977 | *KCTD15* | G | 0.65 | 1.54 (1.10-2.14) | 0.58 | 0.67 | 0.91 (0.70-1.19) | 0.50 | 1.17 (0.70-1.96) | 0.54* |
| rs17782313 | 18 | 56002077 | *MC4R* | C | 0.28 | 1.23 (0.85-1.76) | 0.27 | 0.29 | 1.05 (0.80-1.37) | 0.72 | 1.11 (0.89-1.38) | 0.34 |
| rs10838738 | 11 | 47619625 | *MTCH2* | G | 0.36 | 1.21 (0.86-1.68) | 0.28 | 0.35 | 0.97 (0.75-1.26) | 0.83 | 1.05 (0.86-1.29) | 0.62 |
| rs2815752 | 1 | 72585028 | *NEGR1* | A | 0.59 | 1.01 (0.73-1.39) | 0.95 | 0.61 | 1.26 (0.97-1.63) | 0.08 | 1.15 (0.94-1.41) | 0.16 |
| rs10913469 | 1 | 176180142 | *SEC16B* | C | 0.22 | 1.55 (1.04-2.30) | 0.03 | 0.21 | 0.88 (0.65-1.19) | 0.40 | 1.15 (0.87-1.99) | 0.62* |
| rs7498665 | 16 | 28790742 | *SH2B1* | G | 0.38 | 1.23 (0.88-1.71) | 0.22 | 0.40 | 0.95 (0.73-1.23) | 0.68 | 1.05 (0.83-1.36) | 0.64* |
| rs6548238 | 2 | 624905 | *TMEM18* | C | 0.87 | 0.92 (0.57-1.47) | 0.72 | 0.85 | 0.90 (0.64-1.28) | 0.56 | 0.91 (0.69-1.20) | 0.51 |
